# Feasibility, adherence and usability of an observational digital health study built using Apple’s ResearchKit among adults aged 18–84 years

**DOI:** 10.3389/fdgth.2025.1520971

**Published:** 2025-04-29

**Authors:** B. Brady, S. Zhou, D. Ashworth, L. Zheng, R. Eramudugolla, K. J. Anstey

**Affiliations:** ^1^ School of Psychology, The University of New South Wales Sydney, Kensington, NSW, Australia; ^2^ Neuroscience Research Australia, Randwick, NSW, Australia; ^3^ UNSW Ageing Futures Institute, UNSW Sydney, Kensington, NSW, Australia

**Keywords:** life-course, digital health, mHealth, mobile app, usability

## Abstract

**Objective:**

This study evaluated the Labs Without Walls app and paired Apple Watch devices for remote research among Australian adults aged 18–84.

**Methods:**

The study app, built using Apple's open-source ResearchKit frameworks, uses a multi-timescale measurement burst design over 8-weeks. Participants downloaded the app, completed tasks over 8 weeks, and wore Apple Watch devices. Feasibility was assessed by recruitment, remote consent, and data collection without training. Adherence was measured by task completion rates. Usability was assessed by response times, a post-study survey, and qualitative feedback.

**Results:**

228 participants (mean age 53, age range 18–84; 62.7% female) were recruited nationwide, consented remotely, and provided data. 201 (88.16%) completed the 8-week protocol. Task adherence ranged from 100% to 70.61%. Health, environmental, and sleep data were collected passively. Usability feedback was excellent, with 84% rating the app as “extremely” or “a lot” user-friendly, 88% finding alert frequency “just right,” and 95.7% finding the schedule manageable. Few age or sex differences were found.

**Conclusions:**

The Labs Without Walls app and paired Apple Watch devices are user-friendly and enable adults aged 18–84 to complete surveys, cognitive and sensory tasks, and provide passive health and environmental data. The app can be used without formal training by males and females living in Australia, including older adults. Future iterations should consider gamification and strategies to improve daily-diary survey user experience.

## Introduction

ResearchKit, a software framework developed by Apple, allows researchers to create research apps for iOS devices. It provides a set of tools for building apps that can collect data from participants, such as survey templates, and pre-built cognitive and sensory tasks. ResearchKit easily integrates with HealthKit, allowing researchers to access health data from participants' iPhones and paired Apple Watch devices. Being open-source, researchers can also customise and extend ResearchKit to fit their specific needs. The rise of digital health has fundamentally transformed health promotion, offering innovative avenues for data collection and intervention delivery. Mobile technologies, like those supported by ResearchKit, are pivotal in this transformation, enabling researchers to reach diverse populations and gather rich, real-time data. This context highlights the growing importance of understanding the feasibility and acceptability of digital health tools in research. In this study, we used ResearchKit to create a research app, Labs Without Walls (1), to collect novel data on micro-longitudinal ageing processes among Australian adults aged 18–84.

Micro-longitudinal studies, which involve repeated measurements over various time scales (2–4), are essential for understanding human development across the lifespan. Lifespan developmental theories (5, 6) suggest that human development is a continuous process throughout life, with individual variations in developmental patterns. Mobile technologies, such as smartphones and watches, offer several advantages for conducting these studies, including improved accessibility, engagement, and temporal granularity (7–9).

Evaluating digital health interventions often begins with assessing feasibility, task adherence, and usability (10). These evaluations can identify methodological elements that are acceptable for different participants and contribute to data quality over time. Benchmarks for success can vary widely, influenced in part by wide variation in the nature, intensity and duration of digital health studies. For example, a review of participant engagement in mobile app interventions found an average overall study retention rate of 67.83% from 54 included studies (11). Study retention ranged from 14% in a mental health study among 348 participants across 12 weeks (12) to 100% retention in a weight loss study among 12 participants across four weeks (13).

The acceptability of research apps and wearables, including task adherence and usability, might vary by participant age or sex. While some studies suggest potential differences, the literature lacks evidence on age or sex differences in multi-timescale measurement burst designs among life-course samples over extended periods.

Demonstrating the acceptability of research apps built with ResearchKit is crucial. Despite age-related differences in digital literacy (14), research has shown that older adults can effectively use digital technologies. For example, a review by Wrzus and Neubauer (15) found no clear age-related trend in compliance rates in ecological momentary assessment (EMA) studies, though women were generally more compliant than men. This aligns with research on gender differences in conscientiousness (16). By demonstrating the acceptability of research apps and wearables across the lifespan, researchers can challenge stereotypes and expand the potential reach of research to hard-to-reach populations, including older adults and others who may not usually be included in research.

This study aims to evaluate the feasibility, adherence, and usability of the Labs Without Walls research app (1) and paired Apple Watch devices (Apple Inc) for studying micro-longitudinal processes among Australian adults aged 18–84 over an 8-week period.

We pre-registered the following hypotheses:

• **H1 (Feasibility, Adherence):** Participants aged 18–84 years will be able to be successfully e-consented, able to input survey data through the Labs Without Walls research app, and have passive data collected using an Apple Watch (Apple Inc) over 8 weeks.
• **H2 (Usability):** The user experience of the Labs Without Walls research app and Apple Watch (Apple Inc) will be rated as acceptable by research participants aged 18–years.

## Materials and methods

This study was approved by the University of New South Wales Human Research Ethics Committee (approval number HC200792). The study design and hypotheses were preregistered on May 4, 2022, using Open Science Framework, before completing data collection. The study protocol is published elsewhere (1).

### Participants

228 Australian adults (18–84) participated in the 8-week study using the Labs Without Walls app. Sample size was estimated based on thresholds of.05 (two-tailed) probability of rejecting the null hypothesis and power of.80, and a previous meta-analysis which estimated the odds ratio of subjective age (one of the primary interests of this broader project) impacting overall health to be 1.57 (17). G*Power determined a minimum sample size of 129. We over-recruited to account for covariates and potential attrition. Participants were recruited through social media, mailing lists, and volunteer databases. Eligible participants (aged 18–85, residing in Australia, owning an iPhone, not requiring text-to-speech to use iPhone) were invited to download the app. Non-responders were followed up with three attempts. Informed e-consent was obtained, and explicit permissions were required for passive data collection on health and environmental measures.

### Design

As described in the study protocol (1), the research app was built for iOS using customised templates provided by Apple ResearchKit (Apple Inc). Amazon Web Services was used to host secure back-end data collection. All participants were provided with an Apple Watch Series 5 (Apple Inc) and Apple wired EarPods (Apple Inc) to use for the duration of the study. Participants returned the Apple Watch (but not the EarPods) at the end of the study, with postage paid for by the study team. Over eight weeks, participants completed a multi-timescale measurement burst protocol, including a baseline survey, repeated surveys on COVID-19 experiences, week-long daily survey sprints which explored daily subjective aging and gender expression, repeated game-like cognitive and sensory tasks, and an end of study usability survey. Participants also provided passively collected health and environmental data from the iPhone and Apple Watch (Apple Inc). Following the baseline survey, study tasks were intended to take no more than a few minutes per day to complete. Further details regarding the technical architecture of the app, study tasks and schedule are reported elsewhere (1).

### Outcomes

#### Feasibility

Feasibility was assessed by the ability to remotely recruit a life-course sample, the location of participants indicating the ability to recruit from a wide geographic area, and overall completion rates. Our benchmarks were successful enrolment and retention of adults aged between 18 and 85 years, recruitment from a wider geographical area than traditional lab-based methods, and a completion rate of 68% or higher (11).

#### Adherence

Adherence was assessed by task completion rates and data completeness. Lower completion rates might indicate difficulty completing tasks in the context of their daily lives (18) or declining adherence over time (19). Incomplete health, environmental, or sleep data might indicate less compliance with the study protocol. Our benchmark for task adherence was 60% completion. We did not set specific benchmarks for passively collected data but anticipated higher completeness for daily behaviours and non-optional tasks.

#### Usability

Usability was assessed by task completion times, an end-of-study survey, and qualitative feedback. Our benchmark for task completion times was alignment with estimated times. For the survey, we aimed for 80% positive ratings for study schedule manageability, watch wearability, usability, alert frequency, and setup/charging ease. Qualitative feedback was sought for future iterations.

### Statistics

Descriptive statistics (frequencies and percentages or means and standard deviations) were used to describe the characteristics of the sample, geographic spread of participants, study completion, task adherence, and the amount of health and environmental data collected from participants across study days. Survey and task completion times were presented as a median for the full sample, to avoid skew due to outliers (e.g., where a study survey remained open and incomplete for several hours). Due to a higher number of females than males in the study sample, Independent-samples Mann–Whitney *U*-tests were used to compare males and females on study outcomes. Linear regression analyses explored the relationship between age-in-years and continuous outcomes. Logistic regression explored the relationship between age and binary outcomes. To allow for possible non-linear effects of age, a quadratic age term was entered into each regression model. Pairwise deletion was used to account for missing data. All statistical analyses were completed using SPSS version 27 (20). Qualitative data provided by participants was reviewed and coded according to the topic(s) raised in each comment. Codes were then used to quantify the frequency of mentions of each topic, and illustrative comments were reported verbatim.

## Results

### Participants

As shown in Supplementary Figure S1, 500 participants expressed interest in joining the study between May 2021 and February 2023. Of those, 342 met our inclusion criteria and were invited to download the Labs Without Walls app and join the study. 228 participants provided study data. Sociodemographic characteristics are summarised in Table 1. The sample was more highly educated and included slightly lower rates of White adults than the general Australian population (21).

**Table 1:** Sociodemographic characteristics of the Labs Without Walls sample (*N* = 228).

Sociodemographic characteristic	*n* (%) or mean (SD)
Age in years	53.09 (18.43), range 18 to 84 years
Years of education^a^	17.84 (3.90) range 8 to 32 years
Sex-at-birth	
Female	143 (62.7%)
Male	85 (37.3%)
Gender Identity	
Man	84 (36.8%)
Woman	139 (61.0%)
Non-binary or gender fluid	4 (1.8%)
Another term (no self-description provided)	1 (0.4%)
Race/Ethnicity	
Arab/West Asian	4 (1.75%)
Black	1 (0.44%)
East Asian	15 (6.58%)
Hispanic/Latin American	2 (0.88%)
South Asian	13 (5.7%)
South-East Asian	8 (3.51%)
White/Caucasian	177 (77.63%)
Other Identity: Arab/West Asian and White/Caucasian	1 (0.44%)
Other Identity: Black and White/Caucasian	1 (0.44%)
Other Identity: East Asian and Arab/West Asian	1 (0.44%)
Other Identity: East Asian and South-East Asian	1 (0.44%)
Other Identity: East Slavic and White/Caucasian	1 (0.44%)
Other Identity: Hispanic/Latin American and White/Caucasian	1 (0.44%)
Other Identity: Jewish	1 (0.44%)
Other Identity: Mediterranean/Southern European	1 (0.44%)
Relationship Status	
Married	111 (48.7%)
In a relationship	45 (19.7%)
Single	51 (22.4%)
Widowed	10 (4.4%)
Divorced	11 (4.8%)
Household Income	
<$300 per week	5 (2.2%)
$300 - $575 per week	16 (7.0%)
$576 - $1,075 per week	45 (19.7%)
$1,076 - $1,700 per week	43 (18.9%)
$1,701 - $2,400 per week	34 (14.9%)
>$2,400 per week	67 (29.4%)
Don't know	18 (7.9%)
Employment	
Employed	142 (62.3%)
Unemployed	17 (7.5%)
Retired	69 (30.3%)

^a^ Years of education indicates a sum of the number of years participants reported attending primary school, secondary school, TAFE and/or University.

### Location of participants

Participants were recruited from all but one of Australia's States and Territories, spanning the breadth of the continent. 2.65% joined the study from the Australian Capital Territory, 63.27% from New South Wales, 12.39% from Queensland, 4.87% from South Australia, 1.17% from Tasmania, 8.85% from Victoria, and 4.87% from Western Australia. Participants were mostly located in urban centres or regional coastal areas, reflecting Australia's population density.

### General study completion

201 participants completed the Day 56 sprint survey, suggesting an overall study completion rate of 88.16%. A logistic regression analysis was conducted to examine the effect of age and its squared term on the likelihood of completing the final study day. Note, the Wald statistic reported below, calculated as the square of the ratio of the regression coefficient to its standard error, is used in association with *p* values to assess the statistical significance of the predictor variable (in this case, age). Neither age in years (B = 0.116, SE = 0.070, Wald = 2.754, *p* = .097, OR = 1.123), nor the age-squared term (B = -.001, SE = 0.001, Wald = .779, *p* = .377, OR = .999) were significant, suggesting that there was no linear or non-linear effect of age on likelihood of completing the day 56 survey. Males and females did not differ in the proportion who completed the Day 56 survey, *χ*2 = 2.44, *p* = .118.

### Adherence

#### Surveys, cognitive and sensory tasks, and sprints

**Figure 1:**
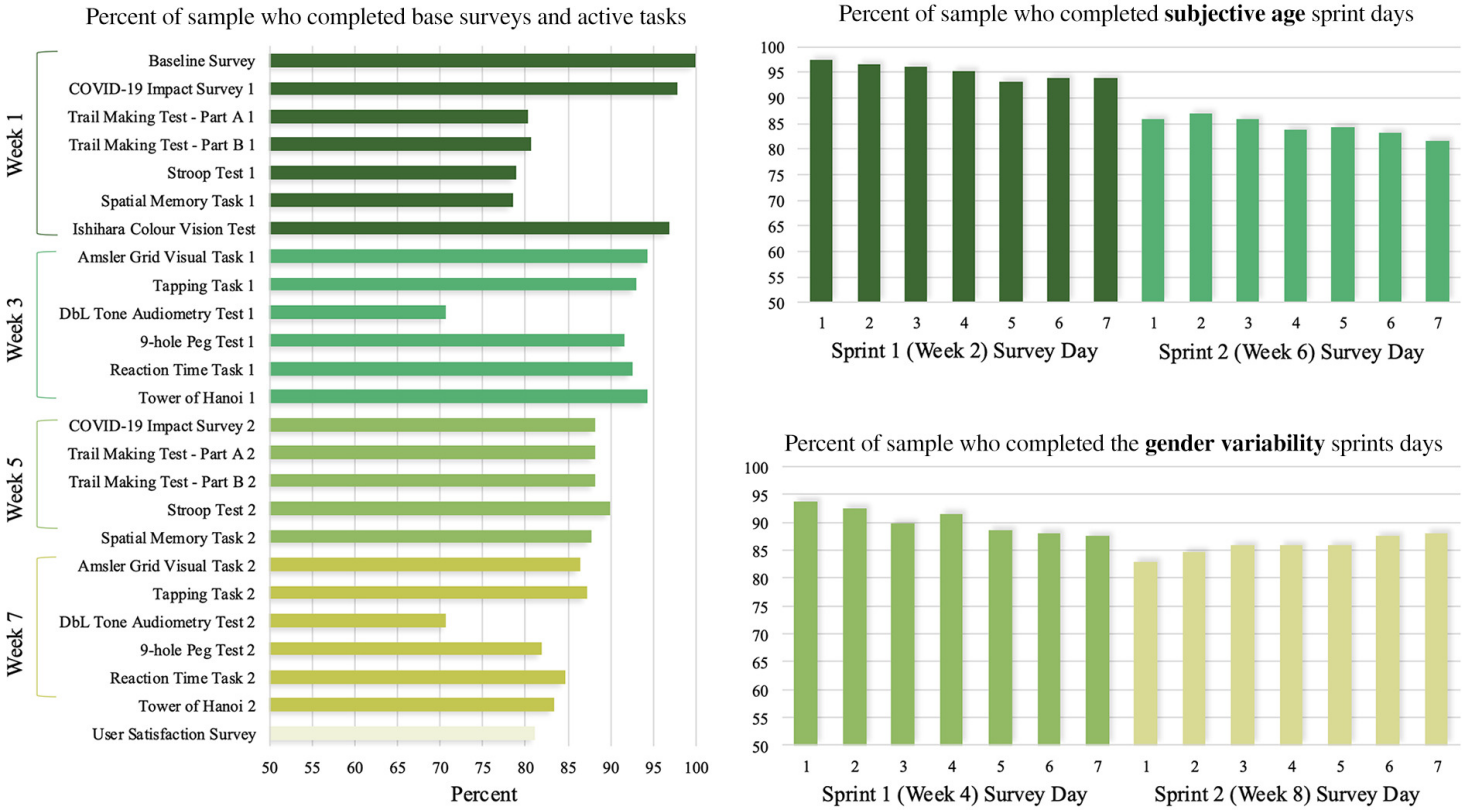
Percent of Labs Without Walls Sample (*N* = 228) who completed each survey, cognitive task, sensory task, and sprint.

Figure 1 shows the percentage of the sample who completed each survey, cognitive and sensory task and sprint day. Adherence ranged from 100% for the baseline survey, to 70.61% for each of the Tone Audiometry tests.

#### Apple watch, health, and environmental data

**Figure 2:**
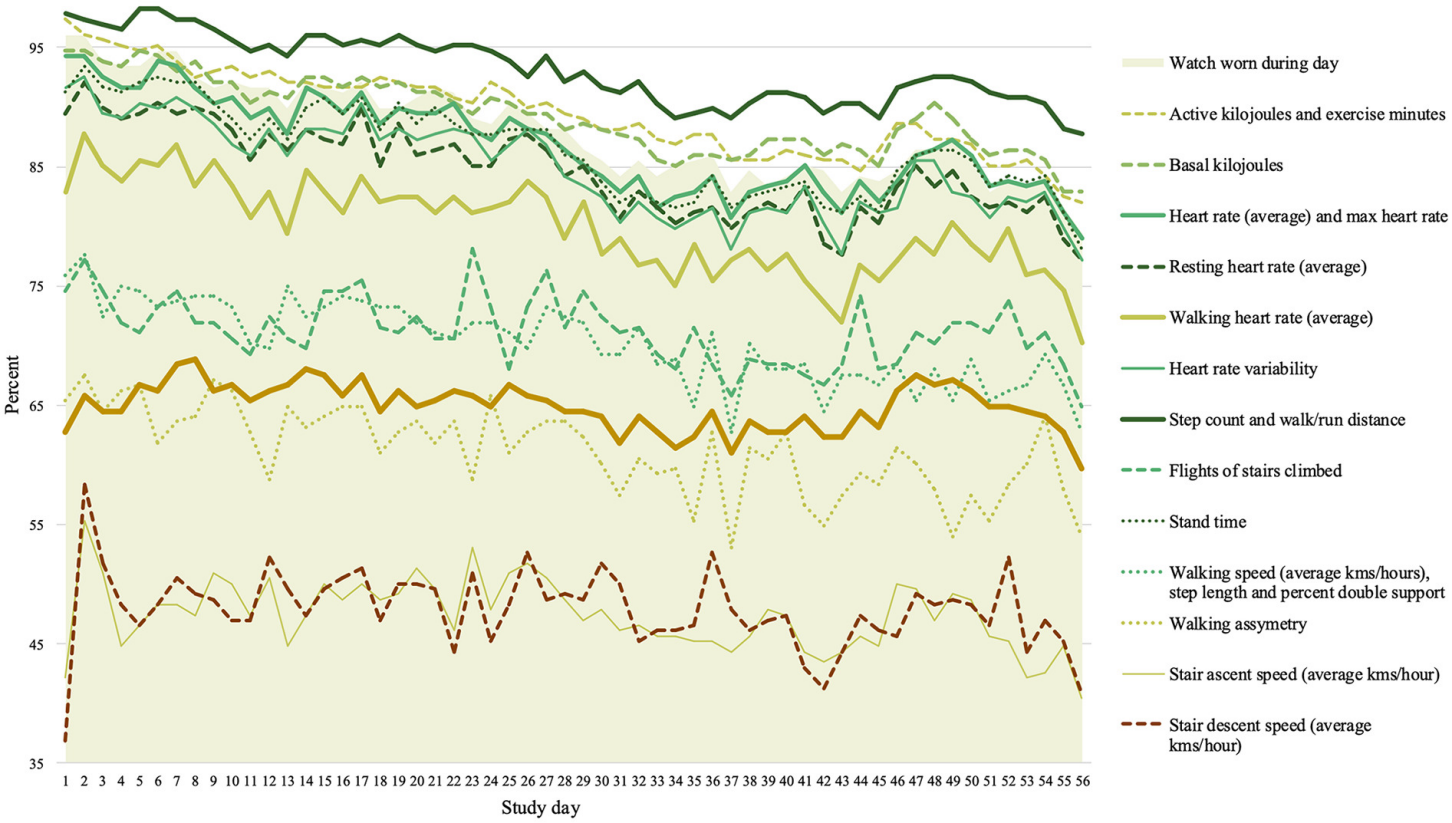
Percent of the total Labs Without Walls sample (*N* = 228) who wore the Apple Watch and provided health and environmental data per study day.

The percentage of the sample who wore an Apple Watch (Apple Inc) and provided health and environmental data is presented in Figure 2. Watches were worn for a median of 55/56 days (range 0 to 56 days) and for an average 16.76 h (SD = 5.38) on the days worn. Over the course of the 8-week study, the percentage of the sample who wore the watch each day fluctuated from 96.05% (*n* = 219) on Day 1, to 79.39% (*n* = 181) on Day 56.

Independent-samples Mann–Whitney *U*-tests revealed that males and females did not differ in the median number of days that the watch was worn, standardised U = 1.844, *p* = .260, or the median number of hours that the watch was worn per day, standardised U = −.013, *p* = .989.

Separate linear regression analyses were conducted to explore the relationship between a) total number of days the watch was worn, and b) total number of hours worn per day and age, including a quadratic term to account for potential non-linear effects of age. Only the regression model predicting total number of days the watch was worn was significant, F(2, 227) = 15.714, *p* < .001, and accounted for approximately 12.3% of the variance in user satisfaction (R^2^ = .123). Age in years was a significant predictor, *β* = 1.097, t(227) = 3.936, *p* < .001. The positive coefficient suggests that higher age was associated with higher number of days wearing the Apple Watch. The quadratic age term was also significant, *β* = −0.009, t(227) = −3.200, *p* = .002, indicating a non-linear relationship between age and number of days wearing the watch showing that the positive relationship between age and days of wear diminishes at higher ages (see Supplementary Figure S2). Age did not predict the average number of hours that the watch was worn each day.

#### Sleep tracking

132 participants provided sleep tracking data. Excluding those who did not provide sleep data, sleep was tracked for a median of 7.00 nights (M = 8.59, SD = 7.13, range 1–36 nights). Independent-samples Mann–Whitney *U*-tests showed that males and females did not differ in the median number of days that sleep was tracked, standardised *U* = .319, *p* = .750. Linear regression was conducted to explore the relationship between the number of nights that sleep was tracked and age. Neither age in years [*β* = −.181, *t*(227) = −1.257, *p* = .210] or the quadratic age term [*β* = .001, *t*(227) = .810, *p* = .419] were significant.

#### Usability

**Figure 3:**
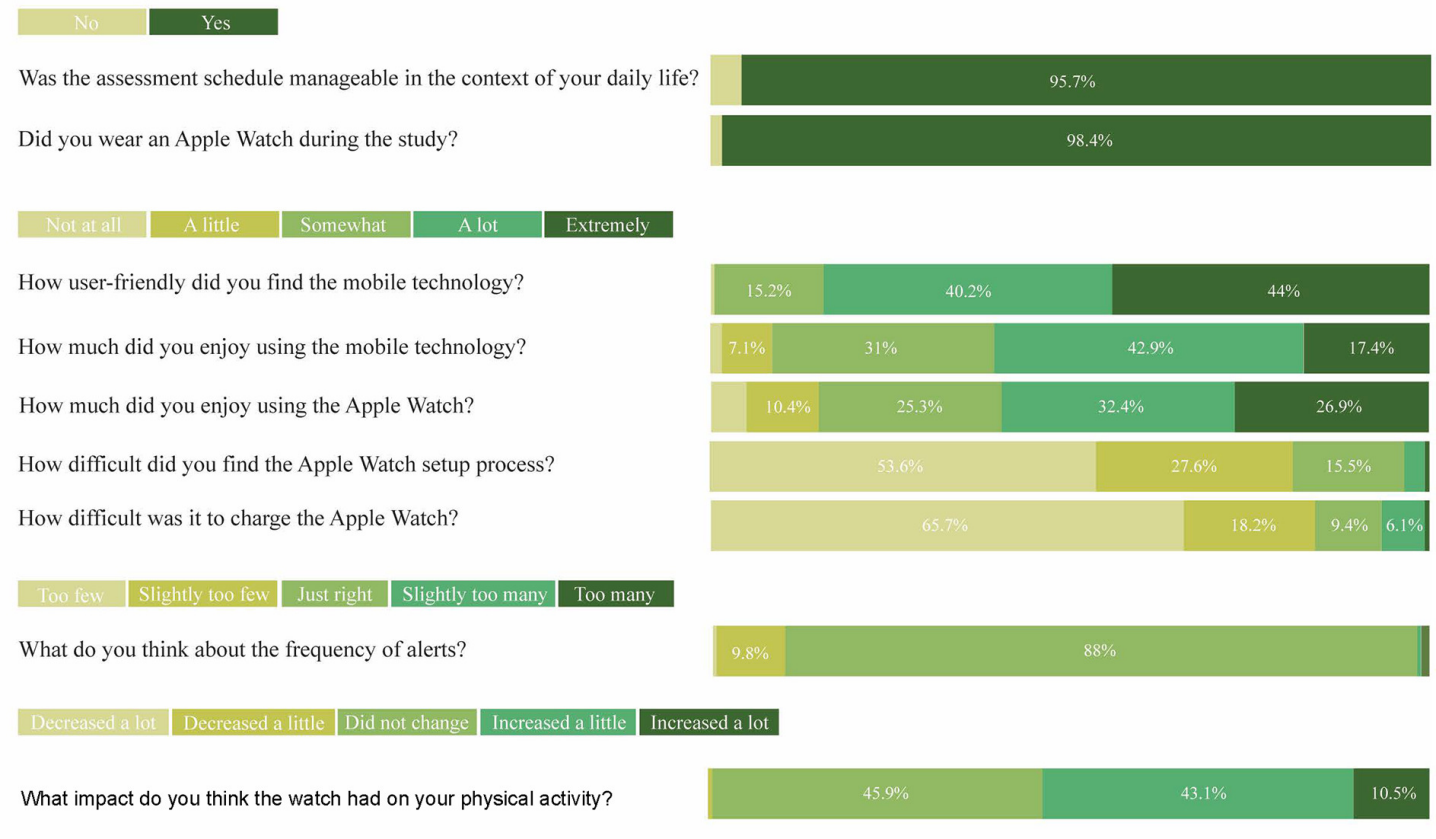
Usability feedback on the Labs Without Walls study, as a percentage of participants who endorsed each response option.

Median completion times for surveys and active tasks were as expected (see Supplementary Table S1). 183 participants started the optional end of study usability module, and 180 provided complete data regarding usability. Figure 3 summarizes the usability feedback. Most of the sample reported that the assessment schedule was manageable in the context of their daily life, and that they wore an Apple Watch (Apple Inc) during the study. Due to a lack of variability in these responses, we were not able to look for age or sex differences in these variables.

As shown in Supplementary Table S2, Independent-Samples Mann–Whitney *U*-tests showed that the distribution of responses to usability questions were the same for males and females for most items rated. However, males and females differed in the frequency of responses to two items: The comfort of the Apple Watch (which was rated as slightly more comfortable by a higher proportion of males), and difficulty charging the Apple Watch (which was rated as marginally more difficult by males).

Separate linear regression analyses were conducted to explore the relationship between usability outcomes and age. Only the regression model predicting perceptions of the frequency of alerts was significant, *F*(2, 178) = 7.076, *p* = .001, and accounted for approximately 7.4% of the variance in user satisfaction (*R*^2^ = .074). Age was a significant predictor, *β* = 0.23, *t*(178) = 2.390, *p* = .018 such that higher age was associated with slightly greater satisfaction with the frequency of alerts. The quadratic age term was not significant, *β* = −0.00, *t*(178) = −1.854, *p* = .065. Age did not predict any other usability outcome.

#### Summary of qualitative feedback on the study

122 participants provided an optional typed response when asked if they had any other feedback they would like to share regarding the study. Of those, 24 provided comments on the study devices, including the iPhone and/or Apple Watch (Apple Inc).

11 participants noted difficulty completing tasks or surveys based on device characteristics,

Some tasks were not really suitable for phones with a smaller screen (I have an iPhone 7)

I found the fine motor task didn't work particularly well on my phone for some reason. Really enjoyed everything else though!

Three participants also noted that the Apple Watch required charging more often than expected,

I found the watch ran out of charge a bit often.

12 participants commented on a potential or experienced technical issue. Of the specific technical issues mentioned, five participants experienced minor display issues with the custom keyboards used to respond to some survey questions or active tasks,

The correction button wasn't visible so if a typo occurred the task had to be cancelled & restarted.

Two participants experienced an issue with the sleep tracking,

Not sure why but the sleeping tracker didn't work. I accepted and then later that day it said it was finished.

27 comments mentioned cognitive or sensory tasks. Six participants noted how much they enjoyed the cognitive and sensory tasks,

All the activities were so fun!

However, 13 participants noted difficulty with the Tower of Hanoi (disk stacking) task, or the 9-hole peg task,

I didnt understand what to do in the disc stacking task. Ive done similar things in the past without a problem. But this one had me stumped.

Some tasks required getting the technique using a phone right first (e.g., the pinching and moving the dot).

48 comments addressed the surveys. Most noted was the repetitious daily surveys within the survey sprints, which 37 of those who commented found to be monotonous, irritating, or boring.

Doing the surveys became really repetitive, as they were the same questions for days on end.

A small number of participants provided feedback on specific survey inclusions, such as

…mood options were limited.

I enjoyed it and was intrigued by the two survey questions that involved gender and did not seem to fit with the idea of perceptions of aging.

21 comments offered suggestions for future content inclusions. Suggestions included:

The ability to add contextual comments on days with a scheduled survey or task,

The option to explain some answers may be useful. For example my first hearing test was effected by a fire alarm. Survey results effected yesterday by a reaction to COVID vaccination

The addition of definitions for core constructs of interest,

Some definition of terms would have been helpful in the survey to ensure reporting on what the researcher wants

The ability to pause the schedule or reset the schedule to an earlier point if interrupted for a block of days,

I was in a mobile/internet black spot for 10 days. I would have liked to rewind back to that block to allow me to fully participate

And the provision of personalised results following surveys or tasks,

Results on each of the tests. Ie you do/do not have colour vision issues, hearing is better in left or right.

Two participants lamented the lack of face-to-face contact with the research team. For one participant, the…

Lack of any face to face researcher/subject meeting meant lack of commitment to study.

38 participants shared positive feedback and/or notes of thanks.

## Discussion

This study evaluated the feasibility, adherence, and usability of the Labs Without Walls research app and paired Apple Watches for 8-week remote micro-longitudinal research. We found strong evidence that this approach is feasible and effective. This supports the growing use of Apple's ResearchKit for cost-effective and accessible app-based research [e.g. (7, 22)]. Our ability to remotely recruit a diverse sample of adults across Australia, including older adults, demonstrates the potential of mobile technologies to reach hard-to-reach participants, regardless of age or location, and is in-line with global research showing that digital research participation is acceptable to people of a range of ages [e.g., (23)].

The 88.16% study completion rate exceeds the average reported in a recent review (11). Despite a large sample and intensive 8-week period, strategies like customizable task notifications likely boosted retention. Age and sex did not impact completion rates, contrary to some previous research that has shown higher completion rates among females compared to males (15).

Adherence ranged from 100% for the baseline survey to 70.61% for Tone Audiometry tests. In-line with advice from Broekhuis et al. (18), poorer adherence for these tests may be due to their longer duration and specific testing requirements. However, all tasks exceeded the 60% completion benchmark. While there was a slight decline over 8 weeks, it was less substantial than in other longer studies [e.g., (19)].

As anticipated, completeness of passively collected health and environmental data varied depending on the frequency of the behaviours being measured. Completeness was excellent for many measures across the full 8-week schedule, including greater than 80% completeness on each study day for wearing the Apple Watch (Apple Inc), active and basal kilijoules, resting heart rate, heart rate variability, and step count. Completeness was lower for measures that required dedicated periods of specific activities (e.g., walking speed, walking assymetry, walking heart rate, and stairs climbed). The poorest completeness was seen for stair ascent and stair descent speeds—the least regular of physical activity patterns studied. Future studies should consider the impact of behaviour regularity on missing data in ambulatory assessment studies. The optional sleep week had a 57.89% completion rate, with no age or sex differences. Future studies should consider lower completion rates for optional elements when planning sample sizes.

The usability ratings were extremely positive. 95.7% of participants found the intensive schedule manageable. This is similar to previous research with smaller, age-restricted samples and less intensive testing schedules [e.g., (24)]. The app was rated user-friendly and enjoyable, and 88% found the alert frequency “just right”. Over 98% wore the Apple Watch, with most reporting no setup or charging difficulties. However, improving support for less tech-savvy participants is an opportunity.

A significant proportion of participants reported increased physical activity due to wearing the Apple Watch. While Labs Without Walls was designed as an observational study, providing the watch may have unintentionally influenced behavior. Longer studies may find that there is an initial increase in activity followed by a return to baseline for most people, however, research is needed to explore this hypothesis. Alternatively, future studies could seek to recruit participants who already own and use an Apple Watch into their research studies. Doing so would reduce or remove the novelty associated with the devices which we believe was the reason for the increased activity in the current study, while also lowering the cost of conducting similar research by removing the need for device purchase and/or postage.

We recommend open-ended feedback as a core element of usability testing in future studies. In this study, participant feedback highlighted opportunities for improvement. Among the most prominent constructive feedback was that participants found the repeated daily surveys to be monotonous. In hindsight, this is understandable given that the questions each day were the same for 7 days at a time, with only a brief justification provided to participants for why the questions were being asked. This daily-diary style approach was important to be able to answer our research questions, however, future research may be able to disrupt the perceived monotony by reducing the overall length of daily surveys and providing greater transparency to participants about the purpose of the sprints which may increase their perceived value and thereby decrease boredom. We note that future research would likely benefit from consultation with community members regarding survey and task scheduling and approaches to improving interest in repetitive aspects of research apps prior to launching full scale data collection.

Qualitative feedback also suggested that participants' experiences of the app as well as data quality could be impacted by device screen size and characteristics of certain active tasks. For example, several participants who joined the study with smaller iPhone devices (e.g., iPhones 6 and 7) found some of the game-like tasks difficult to complete on the smaller screens. This appears to be particularly true for tasks that involved using the touch screen function in a precise way (such as the Tower of Hanoi or the Hole Peg task, both pre-built within Apple's ResearchKit). Future remote research that aims to include such active tasks may benefit from recruiting participants who own iPhones with larger screens.

While the open-source resources provided by Apple's ResearchKit lowered the cost to conduct the study (compared to developing an app from the ground up), there were still considerable additional costs including additional iOS development, return-paid postage of study devices, and cloud storage of study data. Now that the Labs Without Walls app is built and validated as an acceptable research tool, it offers a scalable and relatively cost-effective means of conducting high volume remote research.

Finally, we also acknowledge the inherent selection bias in this study which results from offering a research app that is not compatible with Android devices. There were several important considerations that guided the decision to develop the app for iOS and not Android or both. First, iPhones are the most common smartphone device in the Australian market (25). Second, developing apps is expensive, and associated costs can more than double once you consider developing for both iOS and Android. In this case, developing for iOS was the most cost effective option given the availability of open-source ResearchKit tasks (26) which substantially reduced development time—particularly for the game-like cognitive and sensory tasks we administered from the ResearchKit library—and a grant of Apple Watch devices (Apple Inc) which can be more seamlessly integrated with an iOS app. Additionally, there is evidence for response latency differences across operating systems and devices, which is particularly evident among Android devices given the much greater variability in device manufacturers (27). This calls into question the current comparability of performance across devices on some tasks. There is no doubt that technological innovations in the coming years will remediate some of the concerns above and make it more feasible to build and administer research apps that are equivalent across both iOS and Android devices. We believe this will be an important future step in improving the accessibility of app- and wearable-based research.

## Conclusion

This study provides strong evidence for the feasibility, adherence, and usability of the Labs Without Walls research app and paired Apple Watch devices. By addressing challenges and incorporating participant feedback, future research can further enhance the accessibility and impact of app-based studies in the field of aging research.

## Data Availability

The raw data supporting the conclusions of this article will be made available by the authors, without undue reservation.

## References

[1] Brady B Zhou S Ashworth D Zheng L Eramudugolla R Huque MH A technology-enriched approach to studying microlongitudinal aging among adults aged 18 to 85 years: protocol for the labs without walls study. JMIR Res Protoc. (2023) 12(1):e47053. 10.2196/47053 37410527 PMC10360017

[2] Gerstorf D Hoppmann CA Ram N. The promise and challenges of integrating multiple time-scales in adult developmental inquiry. Res Hum Dev. (2014) 11(2):75–90. 10.1080/15427609.2014.906727

[3] Rickenbach M Almeida DM. Micro-longitudinal research in life-span psychology. Res Hum Dev. (2019) 16(2–3):94–106. 10.1080/15427609.2019.1600057

[4] Sliwinski MJ. Measurement-burst designs for social health research. Soc Personal Psychol Compass. (2008) 2(1):245–61. 10.1111/j.1751-9004.2007.00043.x

[5] Baltes PB. Theoretical propositions of life-span developmental psychology: on the dynamics between growth and decline. Dev Psychol. (1987) 23(5):611. 10.1037/0012-1649.23.5.611

[6] Baltes PB Nesselroade JR. Paradigm lost and paradigm regained: critique of dannefer’s portrayal of life-span developmental psychology. Am Sociol Rev. (1984) 49(6):841–7. 10.2307/2095533

[7] Bot BM Suver C Neto EC Kellen M Klein A Bare C The mPower study, Parkinson disease mobile data collected using ResearchKit. Sci Data. (2016) 3(1):1–9. 10.1038/sdata.2016.11 PMC4776701 26938265

[8] Munos B Baker PC Bot BM Crouthamel M de Vries G Ferguson I Mobile health: the power of wearables, sensors, and apps to transform clinical trials. Ann N Y Acad Sci. (2016) 1375(1):3–18. 10.1111/nyas.13117 27384501

[9] Fischer F Kleen S. Possibilities, problems, and perspectives of data collection by mobile apps in longitudinal epidemiological studies: scoping review. J Med Internet Res. (2021) 23(1):e17691. 10.2196/17691 33480850 PMC7864774

[10] World Health Organization. Monitoring and Evaluating Digital Health Interventions: A Practical Guide to Conducting Research and Assessment. (2016). Available online at: https://iris.who.int/bitstream/handle/10665/252183 (Accessed June 01, 2024).

[11] Oakley-Girvan I Yunis R Longmire M Ouillon JS. What works best to engage participants in mobile app interventions and e-health: a scoping review. Telemed E Health. (2022) 28(6):768–80. 10.1089/tmj.2021.0226 PMC9231655 34637651

[12] Pratap A Renn BN Volponi J Mooney SD Gazzaley A Arean PA Using mobile apps to assess and treat depression in Hispanic and Latino populations: fully remote randomized clinical trial. J Med Internet Res. (2018) 20(8):e10130. 10.2196/10130 30093372 PMC6107735

[13] Morrison LG Hargood C Lin SX Dennison L Joseph J Hughes S Understanding usage of a hybrid website and smartphone app for weight management: a mixed-methods study. J Med Internet Res. (2014) 16(10):e3579. 10.2196/jmir.3579 PMC4259922 25355131

[14] Sims T Reed AE Carr DC. Information and communication technology use is related to higher well-being among the oldest-old. J Gerontol Series B. (2016) 72:761–70. 10.1093/geronb/gbw130 27702839

[15] Wrzus C Neubauer AB. Ecological momentary assessment: a meta-analysis on designs, samples, and compliance across research fields. Assessment. (2023) 30(3):825–46. 10.1177/10731911211002995 35016567 PMC9999286

[16] Schmitt DP Realo A Voracek M Allik J. Why can’t a man be more like a woman? Sex differences in big five personality traits across 55 cultures. J Pers Soc Psychol. (2008) 94:168–82. 10.1037/0022-3514.94.1.168 18179326

[17] Westerhof GJ Miche M Brothers AF Barrett AE Diehl M Montepare JM The influence of subjective aging on health and longevity: a meta-analysis of longitudinal data. Psychol Aging. (2014) 29(4):793. 10.1037/a0038016 25365689

[18] Broekhuis M van Velsen L Hermens H. Assessing usability of eHealth technology: a comparison of usability benchmarking instruments. Int J Med Inf. (2019) 128:24–31. 10.1016/j.ijmedinf.2019.05.016 31160008

[19] Pathiravasan CH Zhang Y Trinquart L Benjamin EJ Borrelli B McManus DD Adherence of mobile app-based surveys and comparison with traditional surveys: eCohort study. J Med Internet Res. (2021) 23(1):e24773. 10.2196/24773 33470944 PMC7857942

[20] IBM Corporation. IBM SPSS Statistics for Windows, Version 27.0. New York, NY: IBM Corp (2020).

[21] Australian Bureau of Statistics. 2021 Census Community Profiles: Australia. (2021). Available online at: https://www.abs.gov.au/census/find-census-data/community-profiles/2021 (Accessed June 01, 2024).

[22] Chan YFY Bot BM Zweig M Tignor N Ma W Suver C The asthma mobile health study, smartphone data collected using ResearchKit. Sci Data. (2018) 5(1):1–11. 10.1038/sdata.2018.96 29786695 PMC5963336

[23] Dumbari NM Gever VC. Online vs face-to-face research participation: which do research respondents prefer? Mdooter J Commun Digit Technol. (2025) 2(1):1–7. https://www.mdooterj.com/index.php/mdooterj/article/view/6

[24] Brewster PW Rush J Ozen L Vendittelli R Hofer SM. Feasibility and psychometric integrity of mobile phone-based intensive measurement of cognition in older adults. Exp Aging Res. (2021) 47(4):303–21. 10.1080/0361073X.2021.1883160 33648422 PMC8225552

[25] Statista. Most popular smartphone brands in Australia as of September 2023. (2023). Available online at: https://www.statista.com/forecasts/1370998/most-popular-smartphone-brands-in-australia (Accessed June 01, 2024).

[26] Apple Inc. Apple ResearchKit framework. (2023). Available online at: https://www.researchandcare.org/researchkit/http://researchkit.org/

[27] Nicosia J Wang B Aschenbrenner AJ Sliwinski MJ Yabiku ST Roque NA To BYOD or not: are device latencies important for bring-your-own-device (BYOD) smartphone cognitive testing? Behav Res Methods. (2023) 55(6):2800–12. 10.3758/s13428-021-01626-1 35953659 PMC9918597

