# Supplementary material for: Feasibility, adherence and usability of an observational digital health study built using Apple’s ResearchKit among adults aged 18–84 years
Source: Front Digit Health. 2025 Apr 29;7:1520971. doi: 10.3389/fdgth.2025.1520971 (PMC12069264; doi:10.3389/fdgth.2025.1520971)
Supplement: Supplementary file 1 [file Table1.docx]

**Supplementary Materials**

**Feasibility, adherence and usability of an observational digital health study built using Apple’s ResearchKit among adults aged 18 to 84 years.**

Correspondence to: Dr. Brooke Brady, School of Psychology, University of New South Wales, Sydney, 2052, Australia. [b.brady@unsw.edu.au](mailto:b.brady@unsw.edu.au)

**Supplementary Figure 1.** Recruitment flow diagram for the Labs Without Walls study.
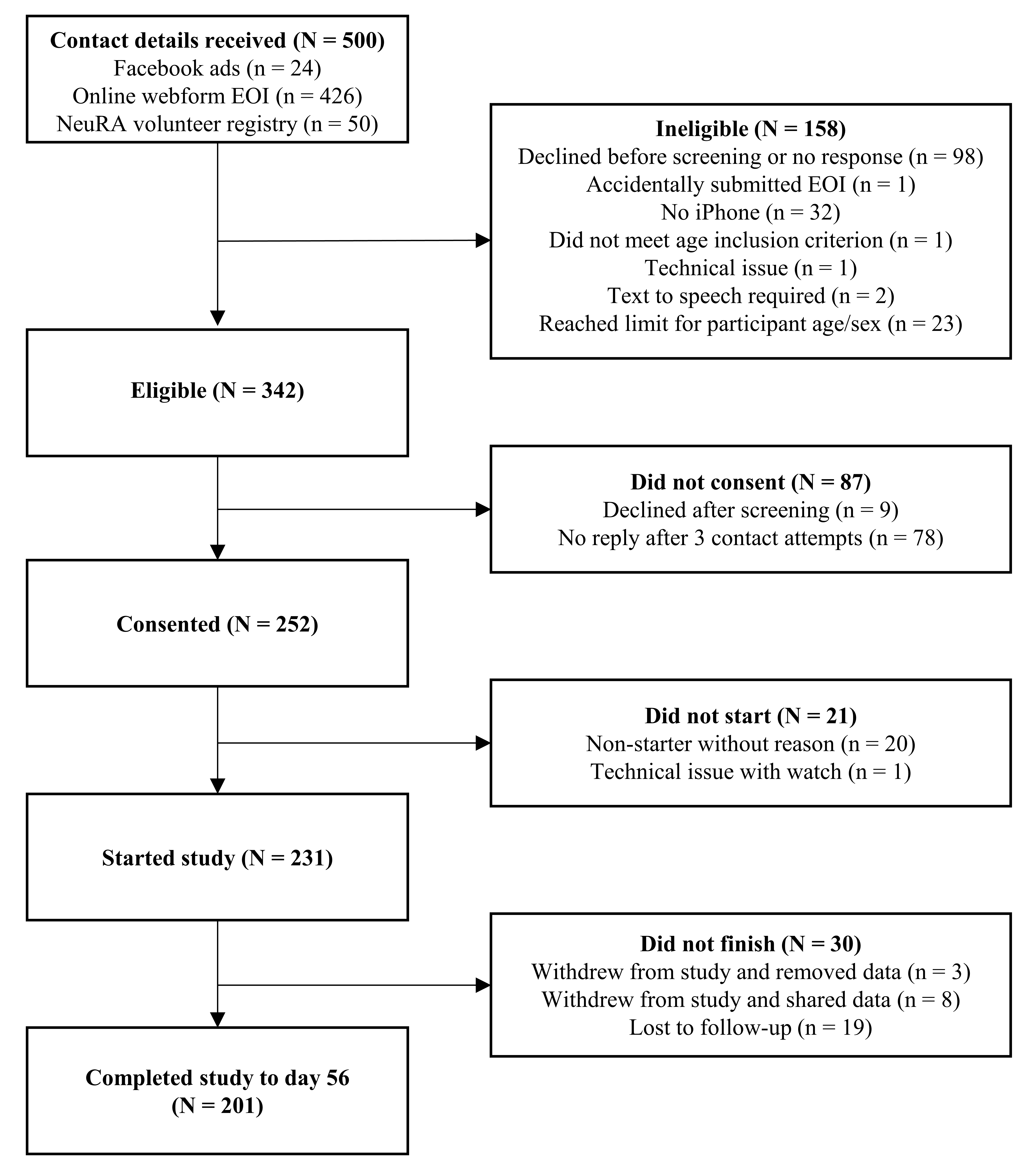


**Supplementary Figure 2.** Bar graph of mean number of days wearing the Apple Watch by age in years.



| Supplementary Table 1. Median Completion Times for Labs Without Surveys and Active Tasks. | | | | | | |
| --- | --- | --- | --- | --- | --- | --- |
| Survey or Task | Study Day(s) | Completion Time (min:sec) |  | Survey or Task | Study Day(s) | Completion Time (min:sec) |
| Baseline Survey | 1 | 13:38 |  | Ishihara Colour Vision Task | 6 | 1:44 |
| COVID Experience Survey 1 | 4 | 5:26 |  | COVID Experience Survey 2 | 30 | 4:48 |
| Subjective Age Sprint 1 Daily Surveys | 8 to 14 | 3:00 |  | Subjective Age Sprint 2 Daily Surveys | 36 to 42 | 2:21 |
| Gender Variability Sprint 1 Daily Surveys | 22 to 28 | 2:00 |  | Gender Variability Sprint 2 Daily Surveys | 50 to 56 | 1:40 |
| Trail Making Task - Part A 1 | 3 | 0:51 |  | Trail Making Task - Part A 2 | 31 | 0:52 |
| Trail Making Task - Part B 1 | 3 | 0:41 |  | Trail Making Task - Part B 2 | 31 | 0:43 |
| Stroop Task 1 | 4 | 2:06 |  | Stroop Task 2 | 32 | 2:08 |
| Spatial Memory Task 1 | 5 | 1:11 |  | Spatial Memory Task 2 | 33 | 1:18 |
| Tapping Task 1 | 16 | 0:49 |  | Tapping Task 2 | 44 | 1:04 |
| Tone Audiometry 1 | 17 | 12:24 |  | Tone Audiometry 2 | 45 | 11:35 |
| 9-Hole Peg Task 1 | 18 | 1:37 |  | 9-Hole Peg Task 2 | 46 | 1:56 |
| Reaction Time Task 1 | 20 | 1:03 |  | Reaction Time Task 2 | 48 | 0:56 |
| Tower of Hanoi Task 1 | 21 | 1:41 |  | Tower of Hanoi Task 2 | 49 | 2:03 |
| Amsler Grid 1 | 19 | 1:16 |  | Amsler Grid 2 | 47 | 1:11 |
| User Satisfaction Survey | 57 | 3:51 |  |  |  |  |

| Supplementary Table 2. Sex Differences (Males, Females) in Frequency of Responses to Labs Without Walls App and Apple Watch Useability Questions. | | | | | | | | | | |
| --- | --- | --- | --- | --- | --- | --- | --- | --- | --- | --- |
| Item | Sex | Not at all | A little | Somewhat | A lot | Extremely |  | Standardized Test Statistic | *SE* | *p* |
| How user-friendly did you find the mobile technology? | Males | 0 | 0 | 8 | 30 | 25 |  | 0.695 | 1.695 | 2.695 |
|  | Females | 0 | 1 | 19 | 42 | 56 |  |  |  |  |
| How much did you enjoy using the mobile technology? | Males | 0 | 1 | 22 | 32 | 8 |  | -0.277 | 322.656 | 0.781 |
|  | Females | 3 | 12 | 33 | 46 | 24 |  |  |  |  |
| How difficult did you find the watch setup process? | Males | 38 | 15 | 8 | 2 | 0 |  | 0.921 | 304.636 | 0.357 |
|  | Females | 58 | 34 | 20 | 2 | 1 |  |  |  |  |
| How much did you enjoy wearing the watch? | Males | 1 | 4 | 15 | 25 | 18 |  | 0.921 | 323.748 | 0.135 |
|  | Females | 7 | 15 | 31 | 32 | 30 |  |  |  |  |
| How comfortable did you find the Apple Watch? | Males | 1 | 4 | 8 | 33 | 17 |  | -2.095 | 320.235 | 0.036 |
|  | Females | 4 | 12 | 35 | 37 | 27 |  |  |  |  |
| How difficult was it to charge the watch? | Males | 35 | 17 | 6 | 4 | 1 |  | -1.994 | 283.144 | 0.046 |
|  | Females | 82 | 16 | 11 | 6 | 0 |  |  |  |  |
|  |  |  |  |  |  |  |  |  |  |  |
|  |  | Too few | Slightly too few | Just Right | Slightly too many | Too many |  |  |  |  |
| What do you think about the frequency of alerts? | Males | 0 | 5 | 58 | 0 | 0 |  | -0.375 | 193.416 | 0.708 |
|  | Females | 0 | 13 | 101 | 1 | 2 |  |  |  |  |
|  |  |  |  |  |  |  |  |  |  |  |
|  |  | Decreased a lot | Decreased a little | No change | Increased a little | Increased a lot |  |  |  |  |
| What impact do you think the watch had on your physical activity? | Males | 0 | 0 | 29 | 27 | 7 |  | 0.021 | 304.529 | 0.983 |
|  | Females | 0 | 0 | 54 | 49 | 12 |  |  |  |  |
| Note. Asymptotic significances are displayed. The significance level is .05. | | | | | | | | | | |
